# Nitric oxide, a survival factor for lens epithelial cells

**Published:** 2008-05-28

**Authors:** Coral G. Chamberlain, Kylie J. Mansfield, Anna Cerra

**Affiliations:** School of Medical Sciences and Bosch Institute, University of Sydney, Sydney, Australia

## Abstract

**Purpose:**

Nitric oxide (NO) is capable of promoting either cell death or cell survival depending on cell type and experimental conditions. In this study, the possible effects of NO on the viability of lens epithelial cells were investigated in an explant model used previously to identify cellular changes associated with posterior capsule opacification following cataract surgery.

**Methods:**

Rat lens epithelial explants prepared from weanling rats were cultured in a serum-free medium for five days with or without the addition of the nitric oxide synthase inhibitor, L-N_ω_-nitro-L-arginine methyl ester (L-NAME), using the inactive enantiomer D-NAME as a control. Alternatively, explants were cultured for nine days with or without the NO donor, sodium nitroprusside. Explants were assessed morphologically and immunohistochemically or by determining DNA content.

**Results:**

In the presence of L-NAME but not in controls, progressive rounding up and detachment of cells from the lens capsule occurred, leading to extensive cell loss. Affected cells showed apoptosis-like cell-surface blebbing and nuclear fragmentation. Conversely, inclusion of sodium nitroprusside suppressed the morphological changes and spontaneous cell loss that occurred when sparsely covered explants were cultured for nine days, increased cell coverage fourfold during that period, and prevented the expression of the transdifferentiation markers α-smooth muscle actin and fibronectin. In addition, whereas L-NAME exacerbated cell loss induced by culturing with 50 pg/ml transforming growth factor-β2, sodium nitroprusside offered protection.

**Conclusions:**

This study points to a previously unidentified role for NO as an endogenously produced survival factor for lens epithelial cells, raising the possibility of using NO deprivation as a means of removing residual lens cells following cataract surgery and thereby preventing posterior capsule opacification.

## Introduction

Many cells have the capacity to synthesize nitric oxide (NO), a readily diffusible, short-lived molecule that is produced by the action of nitric oxide synthase (NOS) on L-arginine. Two of three known cytoplasmic isoforms of NOS, endothelial cell NOS (eNOS/NOS-1) and neuronal NOS (nNOS/NOS-3), are expressed constitutively while the third, inducible NOS (iNOS/NOS-2), is generally expressed in response to immunological challenge or some other pathophysiological stimulus [1,2]. Transient stimulation of constitutive NOS activity results in relatively low levels of NO production whereas iNOS activity can produce much larger amounts of NO over several days [3,4]. A wide variety of biological functions is served by controlled production of NO, which can act both intracellularly as a second messenger and extracellularly as a conveyor of information between cells. However, excessive NO production can result in cellular damage via various mechanisms, which include the formation of highly reactive free radicals such as peroxynitrite [4].

Normal ocular tissues including the retina, ciliary body, iris, conjunctiva, and cornea express NOS [5,6], and NO is normally present at a low concentration in the aqueous humor that bathes the lens [7,8]. Although there is some evidence that constitutive levels of NO production contribute in positive ways to normal ocular function, overproduction of NO in response to induction of iNOS is generally regarded as deleterious. For example, induction of iNOS and abnormal production of NO occur in uveitis, retinitis, and glaucoma [5,6] and in certain animal models of cataract [9-11]. Moreover, the concentration of NO in the aqueous humor is known to be elevated in endotoxin-induced uveitis and traumatic cataract [8,12] and to increase with age in senile cataract patients or following cataract surgery in the rabbit [7,12,13]. A role for NO in the etiology of cataract has been proposed because of its ability to modify lens proteins and/or cause or exacerbate oxidative damage to lens cells or predispose them to such damage [6,14-17].

Lens cells themselves appear to express NOS. NADP-diaphorase activity, which is indicative of NOS activity, has been detected in the rat lens epithelium [18]. In addition, iNOS has been detected at low levels in the normal rat lens by western blot analysis and RT–PCR and shown to be upregulated in the lenses of rats treated with selenite in vivo [9]. iNOS mRNA is also upregulated in human lens epithelial cells cultured with a combination of lipopolysaccharide and interferon-γ [11,19]. Furthermore, it has been shown that opacification of the rat lens in selenite and hereditary cataract models is accompanied by and apparently dependent upon the induction of iNOS in the lens [9,10]. However, little is known about the regulation and biological significance of the synthesis of NO by lens cells under normal conditions.

In other cell types, NO has been shown to affect cell viability in profound and often paradoxical ways. NO may promote either cell death or cell survival in vivo and in vitro depending upon experimental conditions and the tissue or cell type involved. For example, it may either promote apoptosis or protect against apoptosis induced by various means including exposure to transforming growth factor-β (TGFβ) or activation of the Fas pathway [4,20-25]. While progress has been made in elucidating the diverse mechanisms involved in determining cell fate in response to NO, the issues are complex and not yet fully understood. Here, we report the effects of NO on the viability of lens epithelial cells.

## Methods

**Figure 1:**
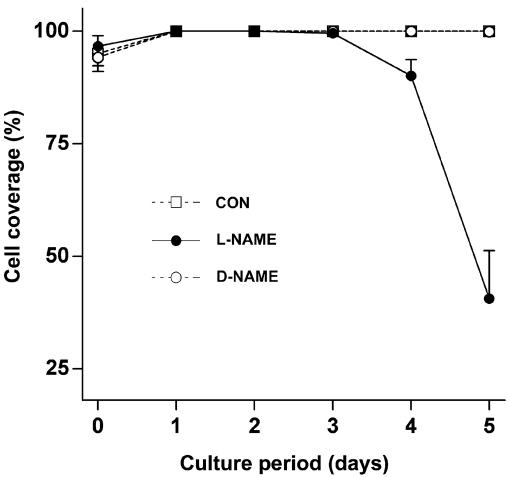
Effect of the nitric oxide synthase inhibitor L-NAME on cell coverage in lens epithelial explants. Explants, initially matched for cell coverage, were cultured in the control medium (CON) or in the control medium supplemented with L-NAME or with the negative control D-NAME at a final concentration of 5 mM. Cell coverage was assessed daily by phase contrast microscopy. Each value represents the mean±SEM of data from 9 to 12 explants. Explants cultured with L-NAME had significantly lower cell coverage than the corresponding control and D-NAME-treated explants on days 4 and 5 (p<0.001, Kruskal–Wallis test with Dunn’s correction for multiple comparisons). NAME stands for N_ω_-nitro-L-arginine methyl ester.

Lens explants were prepared from 20-day-old to 21-day-old Wistar rats as described previously [26,27]. The medium used in all experiments (control medium) was serum-free medium M199 supplemented with 0.1% BSA and antibiotics [28]. All experimental procedures were in accordance with the ARVO Statement on the Use of Animals in Ophthalmic and Vision Research and approved by the Animal Ethics Committee, University of Sydney (Sydney, Australia). HEPES at a final concentration of 20 mM was added to the control medium during initial pinning out of explants. After replacing the HEPES-containing medium with 1 ml of the control medium, explants were examined by phase contrast microscopy and given a ‘cell coverage’ grade ranging from 0%–100%. This was based on an estimate of the percentage of the total explant surface covered by patches of attached, confluent cells [27,29]. Cell coverage immediately after setting up the explants was 57%±3% (mean±SEM). Explants were then assigned to treatment groups matched with respect to initial cell coverage and precultured for one day to ensure uniformly high coverage in all treatment groups at the start of the experiment (day 0; Figure 1). On day 0, the medium was replaced with control medium or with medium containing 5 mM L-N_ω_-nitro-L-arginine methyl ester (L-NAME), a widely used NOS inhibitor, or its inactive enantiomer, D-NAME (both from Sigma, St Louis, MO), readjusted to the control medium pH with 0.5 M NaOH. Previous studies indicate that all NOS isoforms would be inhibited at the concentration of L-NAME used, especially given that lens epithelial cells express esterases capable of converting L-NAME to the more potent inhibitor N_ω_-nitro-L-arginine [30,31]. Explants were cultured at 37 °C in 5% CO_2_/95% air for five days. Cell coverage and morphological changes were monitored daily by phase contrast microscopy. On day 5, explants were fixed as whole mounts in 10% neutral buffered formalin, stained with methylene blue-hematoxylin, and counterstained with Hoechst dye (H33342; Sigma).

Experiments were also performed to test the effects of a classical NO donor, sodium nitroprusside (SNP), on cell survival. Explants with initial cell coverage of 20% or less were used for these experiments because previous studies had shown that sparsely covered explants were susceptible to spontaneous cell loss during culture in the control medium [27]. The explants were assigned to two matched groups and precultured for 24 h as described above and then cultured for nine days with or without the addition of SNP (Calbiochem, La Jolla, CA) at a final concentration of 50 μM. The medium was replaced on day 5 of culture with or without the addition of SNP as appropriate, and explants were monitored daily by phase contrast microscopy. At the end of the culture period, representative explants were fixed as whole mounts in Carnoy’s fixative (3:1 ethanol:acetic acid), and α-smooth muscle actin (αSMA) and fibronectin were immunolocalized by a double-labeling technique with Hoechst counterstaining of nuclei [32].

In some experiments, recombinant TGFβ2 (R&D Systems, Minneapolis, MN) was included in the medium on day 0 with or without L-NAME or SNP and explants were cultured for two days. The final concentration of TGFβ2 was 50 pg/ml, a concentration shown previously to induce rapid loss of cells from rat lens epithelial explants [26,27]. In some experiments, explants were lysed at the end of the culture period and the DNA content was determined by the PicoGreen method as described [33].

## Results

**Figure 2:**
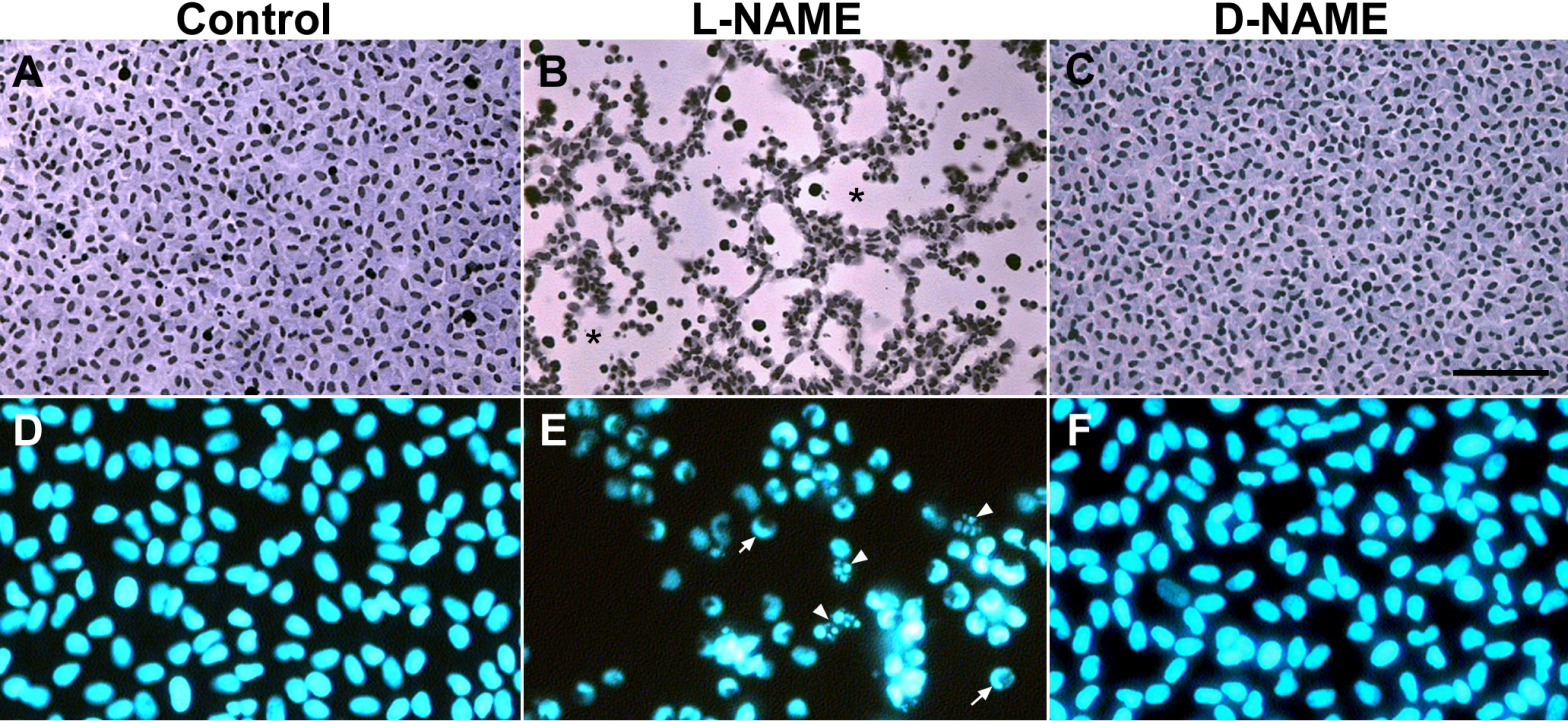
Histology of lens epithelial explants cultured with the nitric oxide inhibitor L-NAME. Explants were cultured in the control medium (**A** and **D**) or in the control medium supplemented with 5 mM L-NAME (**B** and **E**) or D-NAME (**C** and **F**). Representative explants were fixed on day 5 of culture and stained with methylene blue-hematoxylin (**A**-**C**) and Hoechst dye (**D**-**F**). Explants cultured in the control medium (**A**) or the control medium with D-NAME (**C**) were completely covered with a monolayer of closely packed cells in the cobblestone array typical of the normal lens epithelium whereas in the L-NAME-treated explants (**B**) large regions of lens capsule were denuded of cells (denoted by an asterisk). Nuclei in explants cultured in the control medium alone and the D-NAME-treated explants were relatively uniform in shape and evenly stained with Hoechst dye (**D** and **F**) whereas nuclei in the L-NAME-treated explants (**E**) showed crescent-like staining with Hoechst dye (arrow) or nuclear fragmentation (arrowheads). The bar represents 80 μm in **A**-**C** and 35 μm in **D**-**F**. NAME stands for N_ω_-nitro-L-arginine methyl ester.

Over the first three days of culture, cell coverage was virtually 100% in explants cultured in the control medium or in the medium containing L-NAME or D-NAME (Figure 1). However, beyond three days of culture there was a dramatic decrease in cell coverage in explants cultured with L-NAME (Figure 1 and Figure 2B,E). In contrast, explants cultured in the control medium or with D-NAME remained completely covered with a monolayer of cells in cobblestone arrays throughout the entire five-day culture period (Figure 1 and Figure 2A,C,D,F).

**Figure 3:**
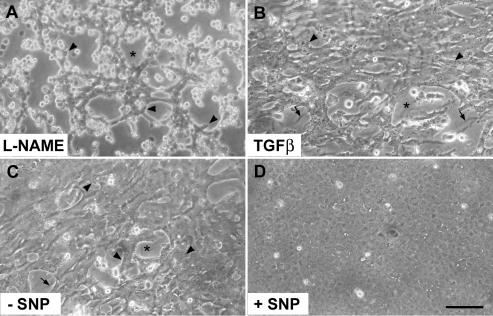
Effects of L-NAME, TGFβ, and SNP on the morphology of lens epithelial explants. Explants were cultured for five days with the NOS inhibitor L-NAME (**A**), for two days with 50 pg/ml TGFβ2 (**B**), or for nine days in the control medium (**C**) or control medium supplemented with the NO donor SNP (**D**) and then photographed by phase contrast microscopy. In explants cultured with L-NAME (**A**), large regions of smooth lens capsule were visible (denoted by an asterisk) between strands of cells and clusters of bright, detached cells, and cells abutting the capsule showed cell-surface blebbing (arrowheads). In explants cultured with TGFβ for two days (**B**) and sparsely covered explants cultured in control medium without SNP for nine days (**C**), progressive cell loss was accompanied by extensive cell-surface blebbing (arrowheads), and exposed regions of the lens capsule (denoted by an asterisk) exhibited wrinkling (arrow). A corresponding explant cultured with SNP (**D**) became well covered with a monolayer of cells in the cobblestone array typical of the normal lens epithelium. The bar represents 80 μm in **A** and 100 μm in **B**-**D**. NAME stands for N_ω_-nitro-L-arginine methyl ester; TGF stands for transforming growth factor; and SNP stands for sodium nitroprusside.

The progressive denuding of the lens capsule that occurred in the presence of L-NAME was accompanied by an extensive loss of cells from the explant. Indeed, 2 of the 11 explants in the L-NAME-treated group were completely devoid of cells by day 5 of culture. During culture, large numbers of cells were observed rounding up, becoming highly refractile, and detaching from the lens capsule. This process was accompanied by cell-surface blebbing, especially at cellular margins abutting denuded regions of capsule (Figure 3A). Nuclear fragmentation, of the type observed in cells undergoing caspase-dependent apoptosis [34,35], was observed in marginal cells in L-NAME-treated explants (Figure 2E) but not in untreated controls or D-NAME-treated explants (Figure 2D,F). The cell loss observed in L-NAME-treated explants (Figure 3A) was comparable in some respects to that observed in explants cultured with 50 pg/ml TGFβ2, although cell loss induced by TGFβ began at an earlier stage of culture and was accompanied by more extensive cell-surface blebbing (see Figure 3A,B). In addition, the capsular wrinkling characteristically induced by TGFβ (Figure 3B) [26,27,32] was not apparent in L-NAME-treated explants (Figure 2B and Figure 3A).

The above experiment suggested that suppressing NOS activity had a negative effect on cell survival. In analogous experiments designed to determine whether the addition of an NO donor promoted cell survival, inclusion of SNP not only promoted cell survival but also permitted the repopulation of the denuded lens capsule. At the end of the culture period, all SNP-treated explants were 50%–100% covered with confluent cells in cobblestone arrays (Table 1; Figure 3D) including an explant with very low initial cell coverage (<5%). In contrast, most explants in the control group cultured without SNP showed extensive and progressive cell loss over the same period (Table 1; Figure 3C).

**Table 1:** Effect of the nitric oxide donor SNP on cell survival and morphology in lens epithelial explants.

**Treatment**	**Without SNP**	**With SNP**	**p-value**
**Day 0**			
Cell coverage (%)	26±6	30±4	NS
Number of explants	8	7	
**Day 9**			
Cobblestone arrays	1	7	0.001
Coverage 50% or more	2	7*	<0.01
Capsular wrinkling	8	1	0.001
Blebbing and cell loss	7	0	0.001

Sparsely covered explants were cultured with or without addition of SNP on day 0 and at change of medium on day 5. Cell coverage values are given as the mean±SEM. Other values indicate the number of explants exhibiting a particular feature. The p-value represents the significance of the difference between explants cultured with or without SNP using Student’s *t*-test to compare cell coverage values and Fisher’s exact test to compare ratios of explants exhibiting a particular feature in the two groups. SNP stands for sodium nitroprusside, and NS stands for not significant. The term “cobblestone arrays” indicates that confluent patches of cells exhibiting the cobblestone array typical of the normal lens epithelium were present. The term “blebbing and cell loss” indicates that explants exhibited cell-surface blebbing and cell detachment. The asterisk indicates that mean cell coverage in this group was 83%±8%.

**Figure 4:**
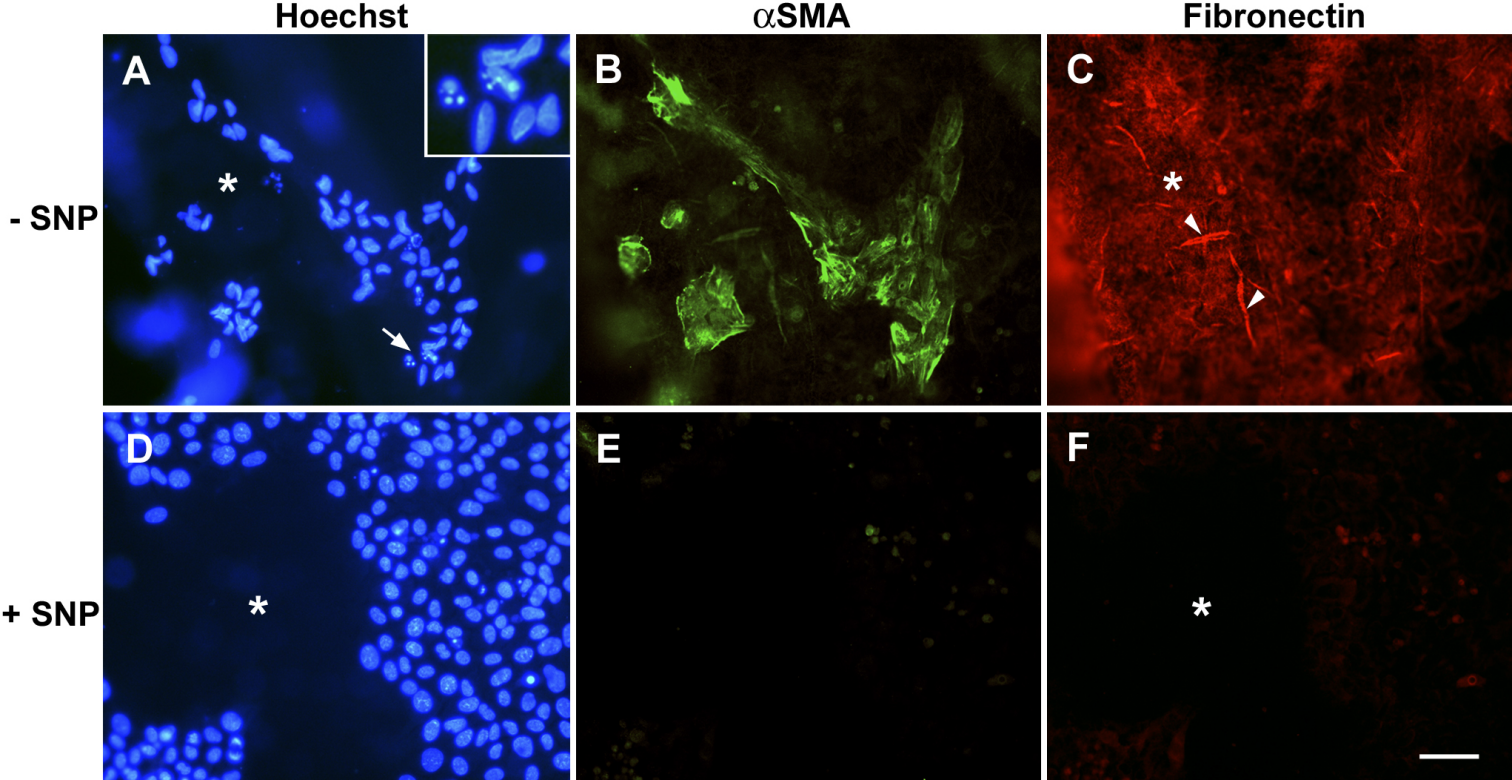
Effect of the nitric oxide donor SNP on immunoreactivity for αSMA and fibronectin. Sparsely covered explants were cultured for nine days in the control medium (**A**-**C**) or in the control medium containing SNP (**D**-**J**). αSMA (**B** and **E**) and fibronectin (**C** and **F**) were then immunolocalized in fixed whole mounts of representative explants by a double-labeling technique with Hoechst counter-staining of nuclei (**A** and **D**). Regions of the lens capsule that became denuded of cells during culture are indicated by an asterisk. The occasional small patches of cells that remained in explants cultured in the control medium alone (**A**) expressed strong reactivity for αSMA (**B**) and fibronectin (**C**). Reactivity for the latter was present throughout the explant including denuded regions of capsule where it was often present in fibrillar form (**C**, arrowheads). The region indicated by the arrow in **A** is given at higher magnification in the inset to show the fragmentation of nuclei. When SNP was included during culture (**D**), specific reactivity for αSMA (**E**) and fibronectin (**F**) was not detectable. The bar represents 50 μm in **A**-**F** and 20 μm in inset in **A**. SNP stands for sodium nitroprusside, and αSMA stands for α-smooth muscle actin.

Including SNP in the medium also suppressed the capsular wrinkling and cell-surface blebbing typically associated with cell detachment under these conditions (Table 1). In the control group, cells abutting the denuded lens capsule were particularly prone to surface-blebbing and detachment (Figure 3C) whereas corresponding cells in SNP-treated explants generally remained bleb-free and extended lamellipodia onto the lens capsule at cellular margins (not shown). Furthermore, residual cells in explants that were cultured in the control medium for nine days exhibited specific reactivity for transdifferentiation markers, αSMA and fibronectin (Figure 4A-C), a finding consistent with previous studies [29,32], whereas reactivity for these markers was negligible in corresponding explants cultured with SNP (Figure 4D-F).

In experiments in which TGFβ2 was included in the medium on day 0, L-NAME appeared to exacerbate TGFβ-induced cell loss during the two days of culture (Table 2A) whereas SNP again exhibited a positive effect on cell survival (Table 2B). The latter was evidenced by the significantly higher cell coverage and DNA content of explants cultured with TGFβ plus SNP compared with those cultured with TGFβ alone (Table 2B). However, the cell-surface blebbing and capsular wrinkling that generally precede cell loss were visible in at least some regions of all explants cultured with TGFβ plus SNP by day 2 of culture, and all these explants became virtually devoid of cells by day 6 (not shown).

**Table 2:** Effects of L-NAME and SNP on cell coverage, morphology, and DNA content of explants cultured with TGFβ.

**A. Treatment**	**TGFβ**	**TGFβ + L-NAME**	**p-value**
Number of explants	8	9	
Cobblestone arrays	1	1*	NS
Cell loss	1	7	0.02

**B. Treatment**	**TGFβ**	**TGFβ +SNP**	**p-value**
Number of explants	5	5	
Cobblestone arrays	1	5	0.05
Cell loss	0	0	NS
DNA (ng/explant)	81±8	123±14	0.03

Matched batches of explants were cultured with TGFβ2 with or without the addition of L-NAME or SNP and were assessed on day 2 by phase contrast microscopy and/or lysing explants and determining DNA. DNA values are given as mean±SEM. Other values indicate the number of explants exhibiting a particular feature. The p-value represents the significance of the difference between explants cultured with or without L-NAME or SNP. Fisher’s exact test was used to compare ratios of explants exhibiting a particular feature in the two groups and Student’s *t*-test to compare DNA values. NS stands for not significant; L-NAME stands for L-Nω-nitro-L-arginine methyl ester, an NO synthase inhibitor; TGF stands for transforming growth factor; and SNP stands for sodium nitroprusside, an NO donor. The term “cobblestone arrays” indicates that confluent patches of cells exhibiting the cobblestone array typical of the normal lens epithelium were present. The term “cell loss” indicates the presence of large regions of lens capsule exposed by cell loss as illustrated in Figure 3B with an overall cell coverage of 50% or less. The asterisk indicates that large numbers of detached, highly refractile cells made it difficult to discern the morphology of the adherent cells in many regions of explants in this treatment group.

## Discussion

The results of this study demonstrate that suppressing NO synthesis by including an NOS inhibitor led to an extensive loss of cells from the lens capsule in initially well covered lens epithelial explants and also exacerbated TGFβ-induced cell loss. On the other hand, inclusion of an NO donor enhanced cell survival in sparsely covered explants under conditions that typically result in spontaneous loss of cells during extended culture and also afforded partial protection against TGFβ-induced cell loss. Therefore, the results point to a previously unrecognized role for NO as a survival factor for lens epithelial cells.

Detachment of cells from rat lens epithelial explants cultured with TGFβ is preceded by changes typical of apoptosis including DNA fragmentation, cell-surface blebbing, nuclear pyknosis, and nuclear fragmentation [36,37]. Cell-surface blebbing and nuclear fragmentation were observed in well covered explants undergoing cell loss in response to L-NAME. They were also observed in sparsely covered, untreated explants undergoing spontaneous cell loss, and in the latter case, these changes were suppressed by including SNP in the medium. Therefore, it is likely that NO was, at least in part, regulating lens epithelial cell survival in these explants by influencing apoptotic pathways as is known to occur for many other cell types [4,20,21,38].

Several growth factors and cytokines have been shown previously to serve as survival factors for lens epithelial cells and/or protect them from apoptosis induced by serum starvation or exposure to agents such as TGFβ and staurosporine. These include FGF [32,39-41], lens epithelium-derived growth factor [42], transferrin [43], insulin-like growth factor-1 [44], and growth arrest-specific gene 6 [45]. It has also been reported previously by others that culturing sparsely covered rat lens epithelial explants in serum-free medium is associated with poor cell survival [46], a finding consistent with the present study (Table 1 and Figure 3C) and a previous study in this laboratory [27]. The data of Ishizaki et al. [45] indicated that lack of diffusible survival factor(s) produced by the lens cells themselves contributed to cell death when cell numbers were low. They also showed that FGF-2 was ineffective as a substitute for this survival factor. Furthermore, it has recently been reported that lens epithelial cells are capable of producing unidentified diffusible factor(s) that protect them against Fas-induced apoptosis provided that they remain attached to the lens capsule or a collagen substratum [47].

The finding of the present study that cell loss is largely prevented in sparsely populated lens epithelial explants by including a source of NO strongly suggests that cell death may occur because of depletion of endogenously produced NO when few lens epithelial cells are present. Moreover, because lens epithelial cells express TGFβ and experience autocrine TGFβ stimulation following wounding of the epithelium [48,49], cell death that is initiated by NO deficiency in lens epithelial explants may be linked with or augmented by TGFβ-induced apoptosis. In lens epithelial cells, the latter process is known to be associated with myofibroblastic/fibroblastic transdifferentiation [50-52]. Thus, the finding that spontaneous cell loss during long-term culture of sparsely covered explants resulted in the induction of transdifferentiation markers is consistent with the suggestion that stimulation by endogenous TGFβ accompanies loss of cells due to NO deprivation. Furthermore, transdifferentiation as well as cell loss were suppressed by including an NO donor.

NO has been shown to have the potential to exert either harmful or beneficial effects in other cellular systems via a variety of mechanisms [4,20,21,53]. These include both pro-apoptotic and anti-apoptotic effects. The outcome depends not only on the concentration of NO to which the cells are exposed but also on factors such as their capacity to deal with oxidative stress and the relative abundance of other free radicals in their environment. Generally, anti-apoptotic effects are observed at relatively low concentrations of an NO donor (30–300 μM SNP) whereas the induction of apoptosis requires higher concentrations (1–4 mM SNP) [54-60], although exceptions to this general rule have been noted (see reference [61], for example). At high concentrations and under conditions that lead to the generation of large amounts of peroxynitrate, NO is cytotoxic and may induce cell death by necrosis if not by apoptosis [38]. The paradoxical anti-apoptotic and pro-apoptotic effects of NO at low and high concentrations, respectively, can occur in the same cell type [54-57].

Oxidative damage to the lens, especially the lens epithelium, is thought to be a triggering factor in the etiology of several forms of cataract, and NO is regarded as an agent capable of contributing to such damage [6,14-17]. Consistent with the latter suggestion, changes that are indicative of oxidative stress and therefore potentially cataractogenic were noted in a study in which rat lenses were exposed in vitro to an NO donor at a high concentration (1 mM) [17]. However, consistent with findings for other cellular systems cited above, exposing explanted lens epithelial cells to an NO donor at a low concentration (50 μM) in the present study promoted lens epithelial cell survival. Furthermore, the low concentration of SNP used in the present study also prevented fibroblastic transdifferentiation, an early event in the etiology of certain forms of cataract and the sight-threatening condition known as posterior capsule opacification, a sequela of cataract surgery [36,50,52,62].

It is not known whether a low level of stimulation by NO is required in the eye in situ to maintain a healthy lens epithelium and protect against cataractous transdifferentiation under physiological or adverse conditions. However, NO is known to be present in the lens environment under physiological conditions. A low concentration of NO is present in the aqueous that bathes the lens anterior. Furthermore, the lens epithelium and neighboring ocular tissues express NOS in situ and therefore represent potential sources of NO ( [5,7-9,18] and see Introduction).

In the experiments reported here, the NOS activity inhibited by L-NAME may have been baseline activity associated with the lens epithelium in situ. However, some damage to the lens epithelium inevitably occurs during the preparation of explants such as those used in the present study, and it is not clear whether the rate of NO production was influenced by such damage. Irrespective of this, the present study has clearly identified a new role for NO as an endogenously produced survival factor for lens cells with the potential to play a role in maintaining the integrity of the lens epithelium.

Human cataract surgery results in extensive damage to the lens epithelium. Many lens epithelial cells are removed by excision of the central anterior capsule and adhering lens epithelium during this procedure. However, variable numbers of lens epithelial cells remain in situ, attached to the annulus of anterior lens capsule that is retained. These cells have the potential to undergo fibroblastic transdifferentiation and migrate posteriorly into the visual axis causing posterior capsule opacification [49,62,63]. The present study raises the possibility of inducing the lens epithelial cells that remain after cataract surgery to die by restricting NO availability through means such as inhibiting NOS production or activity or sequestering NO. Thus, it offers a new perspective on preventing posterior capsule opacification.
